# Role of ethics, meritocracy, and professionalism in public sector reforms: A Q methodology study

**DOI:** 10.1371/journal.pone.0342981

**Published:** 2026-02-20

**Authors:** Munshi Muhammad Abdul Kader Jilani, Samira Tasnim, Nahian Rahman, A S M Rafad Asgar, Nasim Ahmed

**Affiliations:** 1 Department of Human Resource Management, Bangladesh Institute of Governance and Management (BIGM), University of Dhaka (Affiliated), Dhaka, Bangladesh; 2 Research Wing, Bangladesh Institute of Governance and Management (BIGM), University of Dhaka (Affiliated), Dhaka, Bangladesh; 3 Department of Governance and Public Policy, Bangladesh Institute of Governance and Management (BIGM), University of Dhaka (Affiliated), Dhaka, Bangladesh; Joaquim Nabuco Foundation: Fundacao Joaquim Nabuco, BRAZIL

## Abstract

Public sector reforms are crucial for improving governance efficiency, accountability, and service delivery. Their success, however, hinges on key factors such as ethics, meritocracy, and professionalism. This study utilizes Q methodology to explore various perspectives on public sector reforms, drawing on New Public Management (NPM) theory to identify critical priorities and challenges in reform implementation. By employing a structured sorting technique, the study captures stakeholders’ subjective viewpoints in governance and public administration, revealing six distinct perspectives. The findings highlight the vital role of ethical governance (Factor 1) in fostering transparency and public trust while also stressing the importance of strong political will to reduce political interference (Factor 2). Furthermore, the institutionalization of meritocracy and professionalism (Factor 3) is essential for improving administrative efficiency, minimizing bureaucratic inefficiencies, and creating clear career pathways. A collaborative approach that incorporates civil society and private-sector engagement (Factor 4) is also deemed necessary for sustaining reform efforts. However, deeply rooted bureaucratic cultures and institutional resistance (Factor 5) pose significant obstacles to the implementation of reform. Additionally, the study emphasizes the need for continuous learning, leadership development, and adaptive governance strategies (Factor 6) to ensure the long-term sustainability of reforms. The variety of stakeholder perspectives indicates a complex interaction of ethical considerations, political dynamics, bureaucratic structures, and professional development needs. This research contributes to the discourse on public sector transformation by offering policy recommendations for ethical leadership, merit-based policies, and professional capacity building. Future studies should investigate these dynamics across different governance systems to reinforce institutional reforms worldwide.

## Introduction

Public sector reforms play a crucial role in enhancing governance, accountability, and service delivery, particularly in developing countries such as Bangladesh. It is a focal point of policy discussions, especially concerning the integration of ethics, meritocracy, and professionalism as the country strives to improve governance, enhance service delivery, and achieve sustainable development [1]. In this context, ethics, meritocracy, and professionalism are foundational principles that can significantly influence the effectiveness of public administration. Ethics in governance ensures transparency, integrity, and public trust, reducing corruption and maladministration [2]. Meritocracy in the public sector ensures that appointments and promotions are based on competence and qualifications rather than political affiliation or nepotism, leading to increased efficiency and productivity [3]. Professionalism within the public service cultivates a skilled and ethical workforce committed to serving the public interest and implementing policies effectively [4].

Over the past decade, Bangladesh has experienced remarkable economic growth, with its GDP expanding at an average annual rate of 6–7% [5]. However, despite this progress, the public sector continues to grapple with challenges such as corruption, inefficiency, and a lack of accountability, all of which hinder its ability to effectively meet citizens’ needs [6]. These issues are further exacerbated by politicization, nepotism, and weak institutional frameworks, which undermine the principles of ethics, meritocracy, and professionalism [7]. Bangladesh, like many other developing nations, faces challenges in embedding these principles within its bureaucratic structure. The country has undergone multiple waves of public-sector reform aimed at reducing inefficiencies and improving service delivery. However, despite various efforts, issues such as bureaucratic corruption, political interference, and capacity constraints continue to hinder the establishment of an effective governance framework [8]. This study explores the intersection of ethics, meritocracy, and professionalism in the context of Bangladesh’s public administration and assesses their roles in advancing meaningful reforms. The public sector in Bangladesh and similar governance systems frequently struggle with ethical lapses, the absence of a merit-based recruitment and promotion system, and a lack of professionalism among civil servants. Ethical misconduct, including bribery and favoritism, continues to undermine institutional effectiveness and erode public trust in government agencies [9]. The absence of a robust meritocratic system means that recruitment and promotions are often influenced by political favoritism rather than competency, leading to inefficiencies and decreased morale among civil servants [10]. Professionalism in the public sector remains inconsistent, as inadequate training, lack of accountability mechanisms, and weak performance evaluation systems impede the development of a competent and motivated workforce, as mentioned by Islam [11].

While numerous reform initiatives have been implemented, including civil service acts and governance enhancement programs, the persisting challenges indicate systemic gaps in integrating ethics, meritocracy, and professionalism into administrative structures. The lack of empirical studies examining how these principles interact in real-world governance further exacerbates the problem. Thus, there is a critical need to systematically explore these issues, particularly through participatory methodologies such as Q methodology, which enables an in-depth understanding of subjective perspectives among public servants [12]. This research aims to define and understand the diversity of opinions on how ethics, meritocracy, and professionalism shape public sector reforms in Bangladesh using Q methodology. Q methodology is particularly useful for this research as it combines qualitative and quantitative methods to analyze subjectivity in individuals’ viewpoints. By employing this methodology, the study seeks to uncover patterns of thought among civil servants, policymakers, academics, citizens from states, and governance experts regarding the integration of these principles into public administration. The outcomes of the study will help identify commonalities and conflicts in perceptions, guiding future reform efforts to ensure they align with public expectations and administrative realities. The study addresses the following research questions:

RQ1: How do various stakeholders perceive the role of ethics, meritocracy, and professionalism in shaping the effectiveness of public sector reforms?
RQ2: What are the key challenges in embedding ethics, meritocracy, and professionalism in public administration, and how do they impact reform efforts?
RQ3: How do different public administration stakeholders rank and prioritize ethics, meritocracy, and professionalism in the context of reforms, and what policy recommendations emerge from their perspectives?

This study enriches the governance discourse by informing policymakers, bureaucrats, and scholars about the strengths and limitations of reform, and by providing data-driven policy options. It advances governance discourse by offering policymakers, bureaucrats, and scholars data-driven insights. Uniquely applying Q methodology, this study fills a gap in reform literature by capturing the subjective views of civil servants and policymakers, which were often overlooked in prior studies [13], with a focus on corruption indices and efficiency. Its practical recommendations support sustainable governance reforms in Bangladesh and similar contexts. By utilizing Q methodology, this study bridges this gap by capturing nuanced viewpoints and revealing consensus or divergence among stakeholders regarding ethical governance, merit-based systems, and professional standards. Given the growing emphasis on good governance as a prerequisite for sustainable development, the findings of this research will be valuable to policymakers and international development agencies working on public-sector reforms in developing nations.

### Theoretical framework

The New Public Management (NPM) theory emerged in the late 20th century as a leading model for public sector reform, advocating a shift from traditional bureaucracy to a market-oriented, performance-driven, and citizen-focused approach [14,15]. NPM theory also provides a comprehensive framework for understanding and implementing merit-based, ethically grounded reforms in the public sector. A key focus of NPM is the importance of ethics, integrity, and accountability, which are essential for transparent decision-making and help build trust in public institutions for reform. Rooted in efficiency, accountability, and decentralization, NPM seeks to enhance public service delivery by applying private sector management practices, emphasizing performance metrics, cost-effectiveness, and competition [16]. Its vital features are meritocracy, ensuring recruitment, promotions, and evaluations are based on competence rather than political ties [17]. But the success of NPM reforms largely depends on political will, which enables the creation of meritocratic systems that prioritize competence-based recruitment and performance-driven advancement over patronage [18]. NPM also fosters ethical governance by reinforcing transparency, accountability, and integrity through structured mechanisms [15,19]. By prioritizing responsiveness, this theory supports reform efforts that mitigate bureaucratic inefficiencies, enhance institutional performance, and rebuild public trust. Globally, it has demonstrated the potential to transform public administration by embedding ethics, professionalism, and merit-based governance. Additionally, NPM promotes continuous professional development and capacity building by incorporating private-sector techniques that enhance institutions’ skills and capabilities.

NPM provides a valuable framework to understand how ethics, meritocracy, and professionalism shape public sector reform. Its governance model prioritizes efficiency, accountability, and ethical leadership, ensuring that decision-making processes are guided by integrity and transparency [20]. Simultaneously, NPM advances meritocracy by replacing patronage with skill-based recruitment, incentivizing performance, and fostering professionalism through private-sector practices and capacity building [21]. Structural and institutional reforms aligned with NPM aim to decentralize authority and streamline operations, thereby increasing efficiency. To combat corruption and ensure good governance, NPM integrates accountability, transparency, and anti-corruption mechanisms into public management processes [22]. Furthermore, reforming recruitment, promotion, and performance evaluation systems under NPM ensures that decisions are based on merit and results rather than political influence, thus fostering a culture of performance and professionalism. Together, these elements enhance institutional performance and public trust in government institutions.

## Literature review

### Ethics in the public sector

Ethical governance has long been recognized as a foundational pillar of effective public sector reforms, playing a crucial role in fostering transparency, accountability, and citizen-centric administration. Ethics in public administration is widely recognized in academics and policy as essential for preventing corruption, ensuring impartial decisions, and building trust between government and its stakeholders [23]. In public sector governance, such as in the UK and India, ethics serve as a normative framework that protects institutional integrity, promotes fair service delivery, and strengthens the legitimacy of public institutions. As governance becomes more complex, ethics are increasingly vital for tackling challenges such as corruption and boosting public trust [24].

Research confirms, in the global context, ethics’ crucial role in curbing public-sector corruption, showing a strong link between ethical governance and reduced bribery, nepotism, and financial mismanagement [25]. Moreover, Malaysian governments that implement ethics-based training and establish regulatory mechanisms, such as ombudsman offices and whistleblower protections, see significant improvements in transparency, accountability, and public trust [26]. Moreover, ethical governance is tied to institutional legitimacy; failures in ethics lead to diminished public trust and civic engagement. Thus, ethical leadership is vital for restoring legitimacy in public organizations [27]. Alongside its role in mitigating corruption, ethics also shape decision-making at all levels of governance, ensuring that policies align with fairness, justice, and the public interest [28]. Policy decisions in Norway, especially resource allocation and prioritization of social programs, often involve competing interests. Adherence to ethical principles promotes equity and public welfare over narrow political or economic agendas [29]. Therefore, ethics are essential in public sector reforms extending beyond traditional management to emerging governance paradigms influenced by technology [30]. As artificial intelligence and data-driven decision-making increasingly become more prevalent, ethical considerations must adapt to address challenges related to algorithmic transparency, data privacy, and fairness in automated services.

### Meritocracy in public administration

Meritocracy, a key principle of public-sector recruitment, is interpreted differently across cultural and national contexts. While it traditionally emphasizes impartial competition, systemic non-meritocratic factors often create unequal starting points, challenging its core ideals [3]. Empirical studies based on European countries by Suzuki and Hur [31] show that perceived meritocracy is generally higher in private organizations, though this varies by country and region. Robust NPM reforms and merit-based personnel policies typically reduce disparities between the public and private sectors. Similarly, research on Weberian bureaucracy addressed by Cornell et al. [32] highlights that meritocratic recruitment leads to tangible improvements in governance structures. However, merit-based hiring does not always ensure equitable outcomes in the south asian context. For instance, competitive exams have increased representation of educated outsiders while limiting access for lower socioeconomic groups, disproportionately benefiting middle-class applicants. This trend is consistent with Weber [33] observations of the persistence of class-based disparities in meritocratic recruitment within bureaucratic systems.

Beyond equity, meritocratic hiring has a significant impact on both economic performance and administrative efficiency. While enhancing public sector integrity and governance, it may inadvertently limit private sector job creation by attracting talent to the government, potentially raising unemployment and lowering GDP from a USA perspective [18]. Regional disparities further influence the effectiveness of merit-based hiring. While the European Union sees improved efficiency and integrity from meritocratic recruitment, the impact is minimal in North America and Oceania, emphasizing the contextual nature of such reforms [34]. In the Western Balkans, bureaucratic inefficiencies, such as lengthy approval and extensive paperwork, make recruitment costly and ineffective. Streamlining these processes with faster approvals, less paperwork, and digital tools can boost efficiency and support meritocratic governance goals [35].

### Professionalism in governance

Professionalization in the public sector is crucial for improving service delivery, requiring specialized skills and competencies among executives and support staff. Zhang et al. [36] emphasizes organizational learning and transformational leadership in fostering professional discretion, especially in service organizations that often grant greater autonomy than government agencies. Because a well-structured performance management system enhances professionalism by aligning individual efforts with institutional goals, reducing inefficiencies, and encouraging ethical service. Studies in South Africa highlight the role of performance planning, continuous communication, data-driven decisions, and reviews in boosting accountability and efficiency [37]. Additionally, effective talent management is vital for addressing poverty, inequality, and unemployment in developing economies, ensuring a skilled and capable public workforce [38].

In addition to the Indonesian context, individual professionalization and broader governance mechanisms play a crucial role in affecting public service delivery. While organizational commitment, entrepreneurship, and professionalism support good governance, their impact is limited without strong institutional frameworks emphasizing transparency, accountability, and community engagement [39]. Research based on equity theory shows that responsiveness mediates professionalism’s link to satisfaction, with professionalism and timeliness having more substantial indirect than direct effects, as noted by Piatak and Jensen [40]. Ultimately, scholarly literature from Asian and European perspectives underscores that public sector professionalism requires ongoing development, robust governance structures, and talent management to improve service delivery and governance outcomes.

## Study context and participants

Public sector reforms are essential for improving governance, efficiency, and trust in governmental institutions. Many developed and developing countries pursue such reform to strengthen service delivery and accountability. However, the success of these reforms often depends on the principles that guide their implementation. Among these, ethics, meritocracy, and professionalism play a central role in shaping the effectiveness and sustainability of reform initiatives [3]. Ethics fosters integrity, transparency, and trust; meritocracy ensures competency-based recruitment, and professionalism upholds high standards of conduct, efficiency, and dedication in public service roles, thereby contributing to a more capable and responsive administration. Despite their recognized significance, the interplay and relative importance of these principles in driving successful public sector reforms remain insufficiently explored. Gaining insights into policymakers’, civil servants’, and other stakeholders’ views is essential for developing effective policies that balance these values. This study employs Q methodology, a mixed-methods approach integrating qualitative and quantitative techniques, to systematically analyze and categorize diverse viewpoints on the roles of ethics, meritocracy, and professionalism in public sector reforms. By examining how different stakeholders perceive these principles, this research seeks to provide detailed insights into their relative influence on reform outcomes. The findings contribute to the ongoing discourse on public administration by offering empirical evidence on how these values shape governance structures and affect reform success. Ultimately, the research aims to guide policymakers and public administration scholars in developing strategies to integrate ethical governance, merit-based recruitment, and professional standards to enhance the effectiveness of public sector reforms.

### Research method

This study employs Q methodology for data collection and analysis, as outlined by Brown [41] and Younas et al. [42], to explore stakeholders’ subjective views on ethics, meritocracy, and professionalism in public sector reforms. This study reveals how ethics, meritocracy, and professionalism are perceived as reform drivers by capturing diverse governance perspectives. Specifically, it assesses how these principles are conceptualized, valued, and perceived as catalysts for governance improvements and institutional transformation. Q methodology effectively identifies subjective views in small samples [42], offering a contrast to traditional outcome-focused approaches [3]. Though common in social sciences, it is rarely applied in public-sector reforms, where subjective dimensions remain underexplored, as Reddick et al. [16] mentioned. Recent studies underscore Q methodology’s strength in exploring policymaking processes and governance complexities [43]. Given the multi-dimensional nature of governance transformations, there is a need for a methodological insight that effectively captures a broad spectrum of stakeholder perspectives, including those of civil servants, policymakers, and public administration experts. Addressing this gap, this study investigates how ethics, meritocracy, and professionalism are perceived in reform efforts, uncovering consensus and divergence that shape institutional integrity and administrative change. Following Younas et al. [42], this study adheres to a six-step process to ensure rigor and analytical depth:

A. Concourse Development: Collecting a diverse set of statements on ethics, meritocracy, and professionalism in public sector reforms.
B. Q-Set Construction: Select a representative statement set that captures key theoretical debates.
C. Q-Sorting Activity: Participants rank and prioritize statements based on their subjective perspectives.
D. Post-Sorting Activities: Gathering qualitative justifications to contextualize participant reasoning.
E. Q Factor Analysis: Identifying distinct thought patterns by clustering similar perspectives into factors.
F. Interpretation of Results: Analyzing findings within the broader discourse on public sector reforms, ethical governance, and administrative efficiency.

### Ethical declaration

This study examined the roles of ethics, meritocracy, and professionalism in public sector reforms using Q methodology. It did not involve any human subjects, ensuring that all data were anonymized and aggregated to maintain privacy and confidentiality. No personally identifiable or confidential information was accessed, and since there was no human investigation, the risk of sentimental harm was eliminated. Data were likely collected through routine administrative procedures rather than reflecting the subjective views of different individuals. Ethical approval was not required because the study focused on data analysis without direct interaction with human subjects.

### Steps A and B: Concourse and Q-set development

**Fig 1:**
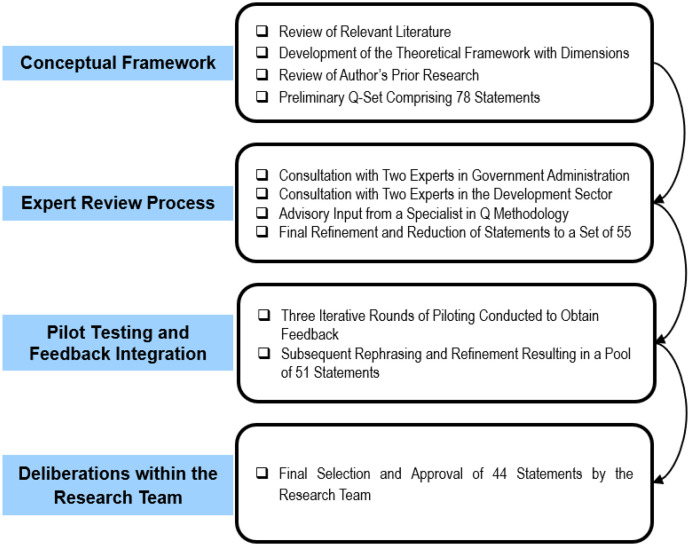
Progression from concourse development to finalized Q-set statements.

This study uses Q methodology to explore ethics, meritocracy, and professionalism in public-sector reforms in Bangladesh. A concourse was developed from existing literature and stakeholder input, then refined through expert review and piloting for clarity and accuracy. This process facilitated the creation of a structured framework for evaluating stakeholder perspectives. The final bilingual 44-item Q-set ensures conceptual precision and participant engagement. This structured approach ensures the study effectively captures stakeholders’ subjective perspectives on the impact of ethics, meritocracy, and professionalism on public sector reforms. Fig 1 depicts the process of concourse development and Q-set construction, while Table 1 shows statement distribution by construct.

**Table 1:** Dimensions and underlying constructs of the Q-set.

Dimensions	Sub-themes	Statement numbers	N = 44
Ethics in the public sector	Ethical framework	1, 3, 5	07
	Training and technological integration	2, 6	
	Anti-corruption strategies	4, 7	
Meritocracy in public administration	Merit-based selection	8, 13, 15	10
	Political influence mitigation	9, 14, 17	
	Transparency enhancement	10, 11, 12, 15	
Professionalism in governance	Strengthening professionalism	18, 23, 24	10
	Skill development	20, 22, 26	
	Minimization of political interventions	21	
	Implementation of a code of conduct	19, 25, 27	
General opinion about the public sector	Transparency and accountability	32, 34, 43, 44	17
	Integration and coordination	29, 36, 38, 39, 41	
	Improving government effectiveness	28, 30, 31, 40, 42	
	Achieving sustainable targets	33, 35, 37	

### Steps C and D: Q-sorting and post-sorting activities

**Fig 2:**
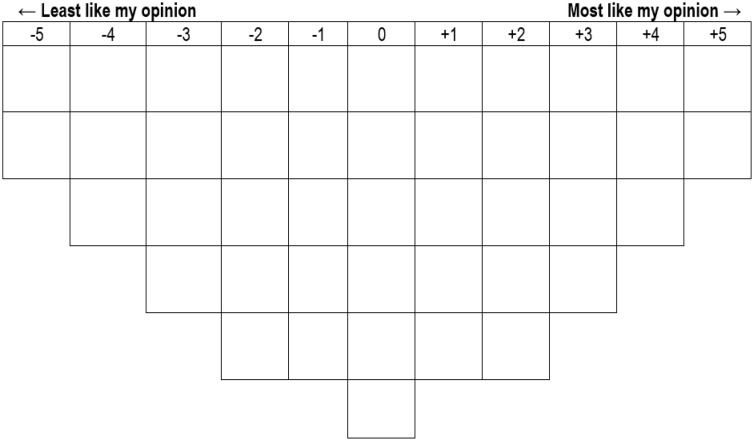
Q-grid displaying a normal distribution format applied to 44 statements.

The Q-sorting and post-sorting activities were conducted during a data collection workshop in November 2024, where participants engaged with paper-based statements and sorting grids. To ensure clarity and procedural consistency, participants received consent forms, written instructions, and an oral introduction to the Q-sorting process, with research team members available for assistance. Of the 121 invited stakeholders, 68 completed the data collection, ranking 44 statements on an unforced-choice distribution grid as shown in Fig 2, ranging from −5 (least like opinion) to +5 (most like opinion) based on their perceived significance. The selection of 68 respondents was determined to ensure a manageable yet diverse sample, capturing a broad range of perspectives across sectors and roles. Stratification was applied to represent key stakeholder groups relevant to the study’s objectives. These rankings allowed participants to organize their perspectives holistically, reflecting their subjective views on governance reforms. After that, a post-sorting activity gathered qualitative insights through open-ended responses, in which participants elaborated on their highest- and lowest-ranked statements and identified additional relevant sources. This enriched analysis thoroughly explains how ethics, meritocracy, and professionalism shape public sector reform.

### Steps E and F: Q factor analysis and interpretation

This study used the KADE tool for Q analysis [44], applying Principal Components and Varimax rotation to determine the most informative factor solution. The factor loadings obtained from the varimax rotation were then used to assess the strength of the relationships between the extracted factors and the representative participant statements, as outlined in S1 Table in S1 File. Higher loading values indicated stronger correlations between participants’ viewpoints and the corresponding factors. For the complete factor loadings for each factor and participants with flagged sorts, please refer to S2 Table in S1 File. Based on qualitative significance, six factors were retained based on eigenvalues ≥1.00, theoretical relevance, and at least two significant loadings per factor [42]. Among 68 participants, 49 loaded significantly (±0.41, p < 0.01) on one of the six factors, while 19 were null and excluded. Five compounded sorts were not classified into specific factors but were included in the analysis. Factor array guided interpretation, supported by demographic and qualitative data, to compare consensus and distinguishing views on how ethics, meritocracy, and professionalism shape public sector reforms.

## Results

### Demographic overview of respondents

The demographic profile of the 68 respondents reflects diverse professional backgrounds across sectors and roles. Table 2 details their age, gender, education, sector, experience, job level, location, political orientation, and stakeholder group. Most participants are aged 41–50 (41.2%) and male (79.4%), with 67.6% holding a Master’s degree. The majority come from the public sector (72.1%), followed by the private sector (20.6%) and the development sector (7.4%). Notably, the sample is predominantly male (79.4%) and comprises primarily individuals from the public sector (72.1%). This composition is well-suited to the study’s focus on public-sector perspectives. Since the research aims to gather viewpoints specific to public institutions, the demographic distribution accurately represents the population being studied. This alignment enhances the study’s contextual relevance without compromising its generalizability. Work experience ranges widely, with 38.2% having 8–14 years of experience and 26.5% having over 22 years. Mid-management roles dominate (57.4%), with fewer senior (29.4%) and entry-level (13.2%) positions. Urban residents constitute 79.4% of the sample. Politically, 61.8% identify as liberal, 29.4% moderate, and 8.8% conservative. Stakeholder representation spans academia (25.0%), ministry of public administration (16.2%), private organizations (16.2%), and other ministries, offering a broad perspective on ethics, meritocracy, and professionalism in public sector reform.

**Table 2:** Demographic overview of respondents (P = 68).

Categories	Details	Frequency	%	Categories	Details	Frequency	%
Participants age	20-30 years	06	8.8	Participants geographical location	Urban	54	79.4
	31-40 years	24	35.3		Suburban	9	13.2
	41-50 years	28	41.2		Rural regions	3	4.4
	51 years and above	10	14.7		Regional department	2	2.9
Gender	Male	54	79.4	Political orientation	Conservative	6	8.8
	Female	14	20.6		Liberal	42	61.8
Level of education	Bachelors	10	14.7		Moderate	20	29.4
	Masters	46	67.6	Interested groups represented in the Q-set	Ministry of Home Affairs	4	5.9
	PhD	12	17.6		Ministry of Health and Family Welfare	4	5.9
Current job sector	Public sector	49	72.1		Ministry of Public Administration	11	16.2
	Private sector	14	20.6		Academician from Universities	17	25.0
	Development sector	5	7.4		Ministry of Finance	5	7.4
Experience in service	0-7 years	12	17.6		Private Organization	11	16.2
	8-14 years	26	38.2		Policymakers from Financial Institutes	4	5.9
	15-21 years	12	17.6		Ministry of Local Government, Rural Development and Co-operatives	8	11.8
	22 years and above	18	26.5		Ministry of Law	2	2.9
Current job position	Entry level	9	13.2		Ministry of Power, Energy, and Mineral Resources	2	2.9
		Mid management	39	57.4			
		Senior management	20	29.4			

### Correlation between factors, participants in each group, and reliability

In Table 3, the factor correlation matrix shows weak interrelationships among the six factors, with correlations ranging from −0.0966 to 0.1073, indicating their relative independence. The low correlation values suggest that these factors are largely independent, supporting their distinctiveness in explaining different dimensions of the study. Factor group sizes vary from five (Factor 2) to eleven (Factor 6), reflecting diverse perspectives. The average reliability coefficient of 0.8 and high composite reliability scores (0.952–0.978) indicate strong internal consistency. Standard errors of factor Z-scores (0.148–0.219) suggest precise factor estimates. These results confirm the robustness and validity of the factor structure in capturing key dimensions of ethics, meritocracy, and professionalism in public sector reforms.

**Table 3:** Correlations between factors, participants in each group, and reliability.

Factors	Factor 1	Factor 2	Factor 3	Factor 4	Factor 5	Factor 6
Factor 1	1.0000	0.0483	0.0249	0.0765	0.0631	−0.0966
Factor 2	0.0483	1.0000	0.0128	0.0115	0.0148	0.0546
Factor 3	0.0249	0.0128	1.0000	0.1021	−0.0174	−0.0529
Factor 4	0.0765	0.0115	0.1021	1.0000	0.0100	−0.0283
Factor 5	0.0631	0.0148	−0.0174	0.0100	1.0000	0.1073
Factor 6	−0.0966	0.0546	−0.0529	−0.0283	0.1073	1.0000
No. of Defining Variables (participants in the group)	8	5	7	8	10	11
Average Reliability Coefficient	0.8	0.8	0.8	0.8	0.8	0.8
Composite Reliability	0.970	0.952	0.966	0.970	0.976	0.978
Standard Error of factor Z-scores	0.173	0.219	0.184	0.173	0.155	0.148

### Factor scores with corresponding ranks and distinguishing Q-sorts

The subsequent sections from the factor analysis resulting in a six-factor solution are presented in Table 4. These factors encapsulate participants’ shared perspectives on the roles of ethics, meritocracy, and professionalism in shaping effective public sector reforms. Each factor is accompanied by a narrative interpretation contextualizing its thematic significance, while representative statements are supported with corresponding quantitative values (provided in brackets). This integrated display provides empirical grounding and interpretive insight into the dimensions respondents perceived as most influential in supporting learning and institutional improvement related to public-sector reform.

**Table 4:** Q-scores and factor z-scores for each item in the Q-sample.

No	Factor 1			Factor 2			Factor 3			Factor 4			Factor 5			Factor 6		
SN	Q-score	z-score	Rank	Q-score	z-score	Rank	Q-score	z-score	Rank	Q-score	z-score	Rank	Q-score	z-score	Rank	Q-score	z-score	Rank
1	3	1.3*	6	0	−0.26	25	1	0.45	16	−4	−1.41	42	−3	−0.97	37	0	0.16	24
2	−1	−0.25	26	1	0.24	18	4	1.42*	3	0	0.17	22	2	0.41	14	−4	−1.4*	40
3	4	1.44	5	3	1.09	8	5	1.9	1	1	0.64	15	−3	−0.98*	38	−5	−2.06*	43
4	1	0.21	18	0	−0.15	22	−5	−1.66	43	−3	−1.03	38	−2	−0.65	33	−4	−1.67	42
5	−5	−1.82	44	−4	−1.58	41	1	0.54*	15	−2	−0.78	35	−2	−0.53	31	−3	−1.15	37
6	3	1.28*	7	−5	−1.64*	43	0	0.28	20	−3	−0.85	36	1	0.28	16	−3	−0.9	36
7	4	1.44*	4	1	0.48	16	1	0.44	17	1	0.59	16	−3	−0.82*	36	0	0.2	22
8	0	−0.07	21	−3	−0.9	38	3	0.94	9	−4	−1.41	41	0	0.11	21	5	1.51	2
9	−3	−1.28*	39	4	1.53	3	2	0.82	13	2	0.81	10	1	0.21	18	−1	−0.27	29
10	−2	−0.61	32	0	−0.13	21	−3	−1.3	39	5	1.57	2	−3	−1.1	39	5	1.78	1
11	−3	−1.13	37	−1	−0.4	27	−1	−0.27	27	3	1.05	7	−4	−1.53	42	3	0.95	8
12	−5	−1.7*	43	−1	−0.32	26	2	0.89*	10	−1	−0.52	29	−2	−0.79	35	−2	−0.51	31
13	−2	−0.87	35	0	−0.18	23	5	1.63*	2	0	0.07	23	2	0.61	11	−1	−0.02	26
14	−3	−1.15	38	3	1.18	7	−4	−1.65	42	−4	−1.41	40	3	1.32	6	1	0.26*	19
15	−4	−1.42	41	−2	−0.71	33	0	−0.13	24	−5	−2.59*	44	0	−0.29	24	2	0.66*	10
16	−1	−0.57	30	1	0.39	17	−1	−0.43	29	−2	−0.64	32	1	0.34	15	−2	−0.87	35
17	3	1.25	8	5	1.85	1	0	0.22	21	1	0.38	19	−5	−1.73*	43	2	0.62	14
18	5	1.84*	1	−3	−0.98	39	3	0.99*	8	−3	−1.31	39	−1	−0.51	30	−1	−0.13	27
19	1	0.47	16	2	0.62	14	−1	−0.22	26	2	0.68	13	4	1.7*	4	0	0.22	20
20	1	0.19*	19	−2	−0.64	32	−2	−0.88	34	−2	−0.56	31	−4	−1.11	40	3	1.12*	6
21	−4	−1.47	42	−5	−1.96	44	−2	−0.74	31	0	−0.18	24	3	0.85	8	2	0.65	11
22	2	0.76	11	2	0.92	10	−4	−1.46*	41	−2	−0.72*	34	0	0.1	22	1	0.27	18
23	−3	−0.95	36	2	0.82	12	0	−0.14	25	0	0.18	21	−1	−0.42	29	−5	−2.42*	44
24	−2	−0.58	31	2	0.91	11	−1	−0.53	30	−2	−0.71	33	1	0.19*	19	4	1.46	3
25	1	0.22	17	−2	−0.6	31	−2	−0.9	35	1	0.55	17	0	0.1	23	0	0.16	23
26	2	0.74	13	−2	−0.72*	34	3	1.29	6	2	0.72	12	3	1.17	7	0	0.22	21
27	0	−0.07	22	5	1.61	2	3	0.99	7	−1	−0.35	26	−1	−0.33	26	−1	−0.22	28
28	4	1.54*	3	−3	−0.83	37	−1	−0.4	28	−1	−0.37	27	0	−0.29	25	−2	−0.63	34
29	5	1.82	2	0	−0.21	24	−2	−0.78	32	2	0.76*	11	5	2.01	2	−1	−0.41	30
30	−2	−0.64*	33	−4	−1.6*	42	4	1.3*	5	0	0.21	20	0	0.14	20	2	0.65	12
31	2	0.8	10	−1	−0.57	28	0	−0.03	23	3	1.05	8	5	2.2*	1	−3	−1.31*	39
32	0	−0.03	20	−1	−0.58	29	−2	−0.82	33	4	1.29*	5	−5	−2.05*	44	−4	−1.43*	41
33	−2	−0.76	34	1	0.24	19	2	0.76	14	5	1.84*	1	−1	−0.4	27	1	0.62	15
34	0	−0.17	25	4	1.44	5	2	0.84	11	2	0.66	14	2	0.56	13	0	0.16	25
35	−1	−0.25	27	−1	−0.59	30	−3	−0.91	36	4	1.51	3	3	0.71	9	3	0.99	7
36	2	0.72	14	−2	−0.74	35	−3	−1.21	37	1	0.43	18	−4	−1.42	41	−3	−1.23	38
37	2	0.75	12	4	1.51	4	−5	−1.72*	44	0	−0.2	25	4	1.49	5	1	0.39	17
38	0	−0.1*	23	3	1.08	9	2	0.82	12	−3	−0.98*	37	4	1.7	3	4	1.43	4
39	−4	−1.38*	40	0	0.04	20	1	0.4	18	3	0.83	9	−2	−0.61	32	−2	−0.6	33
40	−1	−0.4	29	2	0.82	13	−3	−1.23*	38	−1	−0.48	28	−1	−0.41	28	3	0.76	9
41	−1	−0.32	28	−3	−0.8	36	−4	−1.35	40	−1	−0.54	30	2	0.61*	12	−2	−0.55	32
42	3	0.86	9	1	0.49	15	4	1.39	4	3	1.12	6	1	0.26	17	4	1.32	5
43	1	0.49	15	−4	−1.5*	40	1	0.35	19	4	1.45*	4	2	0.62	10	1	0.58	16
44	0	−0.14	24	3	1.31	6	0	0.11	22	−5	−1.5*	43	−2	−0.71	34	2	0.64	13

Note that an asterisk (*) indicates significance at p < 0.01, while all statements shown indicate significance at p < 0.05 and SN: Statement number (Details questionnaire in S1 Table in S1 File).

#### Factor 1: Ethics, integrity, and accountability in public sector reforms.

Factor 1, identified as the most significant dimension with an explained variance of 6%, reflects the critical role of ethics, integrity, and accountability in advancing public sector reforms in Bangladesh. This perspective, strongly supported by eight participants from diverse organizations, underscores the necessity of ethical governance, transparency, and professionalism to foster administrative efficiency. The highest-ranked statement, S-18 (Q-score = 5, z-score = 1.84), highlights a strong consensus that professionalism should take precedence over political loyalty in improving public administration. Similarly, the high ranking of S-29 (Q-score = 5, z-score = 1.82) underscores decentralization as a key strategy for enhancing governance and service delivery. The findings indicate that entrenched corruption remains a formidable challenge to reform, as evidenced by S-28 (Q-score = 4, z-score = 1.54) and S-7 (Q-score = 4, z-score = 1.44), which acknowledge the persistent obstacles posed by political interference and weak oversight mechanisms. The status of ethical governance is further reinforced by S-3 (Q-score = 4, z-score = 1.44), highlighting the symbolic nature of existing ethical codes due to inadequate enforcement. However, the low ranking of punitive measures, such as those proposed in S-5 (Q-score = −5, z-score = −1.82), suggests skepticism about the effectiveness of stringent regulations alone, pointing to the need for broader institutional and cultural transformations. Additionally, meritocratic principles received mixed responses, with S-12 (Q-score = −5, z-score = −1.7) and S-9 (Q-score = −3, z-score = −1.28) ranking negatively, reflecting concerns about the feasibility of merit-based governance amid deeply entrenched, politically motivated appointments. In contrast, the recognition of professional development as a mechanism for fostering accountability and leadership, as reflected in S-20 (Q-score = 1, z-score = 0.19) and S-22 (Q-score = 2, z-score = 0.76), underscores the perceived value of continuous training in enhancing public sector efficiency. Overall, this factor outlines ethics, integrity, and accountability as core pillars of public administration reform while also acknowledging the structural and political barriers that hinder their effective implementation. Sustainable reform strategies should prioritize decentralization, institutional capacity-building, and cultural shifts toward transparency and professional governance rather than relying solely on punitive measures.

#### Factor 2: Political will, meritocracy, and accountability in public sector reforms.

Factor 2, which accounted for 6% of the explained variance and was supported by five participants from diverse organizations, underscores the critical role of political will, accountability, and reduced political interference in advancing public-sector reforms in Bangladesh. The highest-ranked statement, S-17 (Q-score = 5, z-score = 1.85), highlights the necessity of political leadership alongside meritocracy to drive meaningful administrative changes, a sentiment reinforced by S-27 (Q-score = 5, z-score = 1.61), which links public dissatisfaction with governance inefficiencies rooted in entrenched patronage networks and weak oversight mechanisms. The high ratings of S-9 (Q-score = 4, z-score = 1.53) and S-34 (Q-score = 4, z-score = 1.44) further illustrate the perception that political favoritism in promotion practices and resistance to change among public servants impede reform efforts. Additionally, the recognition of salary structure improvements to attract and retain skilled professionals, as reflected in S-37 (Q-score = 4, z-score = 1.51), signifies the importance of financial incentives in enhancing bureaucratic efficiency. However, statements advocating strict ethical codes and harsher accountability measures, such as S-5 (Q-score = −4, z-score = −1.58) and S-21 (Q-score = −5, z-score = −1.96), received lower rankings, suggesting skepticism toward rule-based enforcement without concurrent political and structural reforms. The mixed responses regarding meritocracy, evidenced by the lower rankings of S-8 (Q-score = −3, z-score = −0.9) and S-12 (Q-score = −1, z-score = −0.32), indicate uncertainty about the feasibility of a purely merit-based system within existing political constraints. Conversely, the recognition of ethical lapses in public administration, as highlighted in S-3 (Q-score = 3, z-score = 1.09), suggests an acknowledgment of governance failures that necessitate institutional accountability. This factor underscores the interplay between political leadership and meritocratic principles in enhancing public sector efficiency. The highest-ranked statements emphasize the imperative of strong political will and transparent, merit-based governance structures. The significant positive z-scores associated with statements advocating fair recruitment, merit-based promotions, and performance-driven accountability reflect a consensus on their critical role in driving effective reform. In contrast, the negative z-scores assigned to statements that downplay the necessity of political commitment suggest a prevailing recognition that sustainable public sector reforms require active and sustained political leadership. Overall, this factor emphasizes that while professionalization and meritocracy are essential for public sector reforms, their effectiveness depends on mitigating political interference, ensuring equitable administrative processes, and fostering a culture of accountability and transparency within the governance framework.

#### Factor 3: Strengthening professional development and capacity building.

This perspective, accounting for 6% of the total variance, is significantly associated with seven participants representing academia, policymakers, non-governmental organizations, and various government ministries, underscoring the critical role of meritocracy, professionalism, and the reduction of bureaucratic inefficiencies in public sector reform. This perspective emphasizes the importance of leadership development, continuous training, and ethical governance in enhancing administrative effectiveness while addressing political interference. The highest-ranked statement, S-3 (Q-score = 5, z-score = 1.9), highlights concern regarding weak oversight mechanisms and the symbolic nature of existing ethical codes, underscoring the necessity of institutional accountability. Similarly, the high ranking of S-2 (Q-score = 4, z-score = 1.42) indicates that deficiencies in ethics training and systemic governance weaknesses hinder ethical conduct among public servants, contributing to inefficiencies in service delivery. Furthermore, the strong endorsement of merit-based principles, as reflected in S-13 (Q-score = 5, z-score = 1.63), suggests broad support for inclusive recruitment policies that consider regional and social diversity to enhance fairness and representation within the public sector. The necessity of political commitment to sustaining reforms is also emphasized, as indicated by S-30 (Q-score = 4, z-score = 1.3). At the same time, the importance of professional development in fostering administrative efficiency is evident in the positive rankings of S-26 (Q-score = 3, z-score = 1.29) and S-18 (Q-score = 3, z-score = 0.99), reinforcing the notion that sustained professional growth is essential for governance effectiveness. In contrast, strict punitive measures and rigid accountability mechanisms, as evidenced by the lower rankings of S-5 (Q-score = 1, z-score = 0.54) and S-22 (Q-score = −4, z-score = −1.46), indicate skepticism toward enforcement-based approaches without corresponding institutional and systemic reforms. Notably, financial incentives such as salary reforms were also ranked negatively, as reflected in S-37 (Q-score = −5, z-score = −1.72), suggesting that while professional development is deemed essential, monetary compensation alone is not perceived as the primary driver of public sector professionalism. Factor 3 highlights the significance of professional training, leadership development, and competency-based recruitment in enhancing long-term administrative efficiency. The findings suggest sustainable governance reforms should prioritize institutional capacity-building, ethical oversight, and leadership commitment rather than relying solely on corrective measures or financial incentives. Strengthening professional development initiatives and fostering political will are thus central to advancing public administration reforms in Bangladesh.

#### Factor 4: Structural and institutional reforms for public sector efficiency.

Factor 4, accounting for 6% of the total variance and endorsed by eight participants from various ministries, private organizations, and local authorities, highlights the necessity of policy adjustments, administrative decentralization, and the reduction of bureaucratic inefficiencies to enhance governance effectiveness. This factor underscores the need for a systematic overhaul of governance structures, professional development initiatives, and institutional efficiency to foster sustainable public sector reforms. The highest-ranked statement, S-33 (Q-score = 5, z-score = 1.84), emphasizes a strong consensus on the critical role of professional development programs in enhancing administrative capacity. It is further reinforced by the high ranking of S-35 (Q-score = 4, z-score = 1.51), which advocates aligning public administration reforms with the Sustainable Development Goals (SDGs), reflecting a preference for integrating global best practices and sustainable governance models. A key theme in this factor is the recognition that meritocratic recruitment and structured career advancement are essential to institutional efficiency. The high rankings of S-10 (Q-score = 5, z-score = 1.57) and S-26 (Q-score = 2, z-score = 0.72) suggest strong support for competency-based hiring and promotions to reduce nepotism and foster accountability. While political interference remains a significant concern, as indicated by the positive ranking of S-42 (Q-score = 3, z-score = 1.12), punitive measures aimed at strengthening ethical codes received lower rankings, such as S-5 (Q-score = −2, z-score = −0.78). It suggests that respondents perceive structural reforms as more effective than enforcement-driven approaches in addressing governance inefficiencies. Administrative decentralization and participatory governance were also positively rated, as reflected in S-29 (Q-score = 2, z-score = 0.76) and S-39 (Q-score = 3, z-score = 0.83), indicating a preference for bottom-up governance models that integrate local communities in the reform process. Conversely, statements that downplayed the role of ethics in governance reform, such as S-1 (Q-score = −4, z-score = −1.41), were negatively rated, suggesting prioritization of operational efficiency and institutional restructuring over ethical considerations alone. Overall, Factor 4 strongly emphasizes institutional modernization, administrative streamlining, and structural reforms as the primary drivers of public sector efficiency. The positive z-scores for statements advocating structural changes and professional development indicate consensus on the need for significant institutional reorganization. In contrast, the rejection of statements de-emphasizing institutional reforms reflects dissatisfaction with the status quo. These findings suggest sustainable governance reforms should prioritize capacity-building, strategic institutional planning, and participatory governance while minimizing bureaucratic inefficiencies and political interference.

#### Factor 5: Accountability, transparency, and anti-corruption measures.

Factor 5, which accounts for 6% of the explained variance and includes 10 participants from listed organizations, highlights the critical role of accountability, transparency, and anti-corruption measures in driving effective public-sector reforms in Bangladesh. This factor underscores the significance of localized governance, strong political will, and institutional efficiency in ensuring sustainable administrative transformation. The highest-ranked statement, S-31 (Q-score = 5, z-score = 2.2), affirms that public sector reforms will be ineffective without addressing corruption, emphasizing corruption as a fundamental barrier to governance improvements and reinforcing the necessity of robust anti-corruption strategies. A strong preference for decentralized governance is evident in the high ranking of S-29 (Q-score = 5, z-score = 2.01), which suggests that redistributing power to local governments enhances service delivery and resource management efficiency and is reinforced by the significant agreement with S-38 (Q-score = 4, z-score = 1.7), which advocates for multi-stakeholder engagement, including civil society and the private sector, in the reform process. The role of political interference in undermining governance reforms is also a recurring concern, as indicated by the positive ranking of S-14 (Q-score = 3, z-score = 1.32), which highlights that political favoritism in appointments hinders the recruitment of qualified professionals and weakens the impact of meritocratic reforms. In contrast, statements emphasizing cultural shifts over structural reforms, such as S-32 (Q-score = −5, z-score = −2.05), were rated negatively, suggesting that respondents prioritize concrete administrative and institutional changes over abstract cultural transformations. Views on professional development and career advancement were mixed, with S-26 (Q-score = 3, z-score = 1.17) ranked positively, indicating support for structured career progression to sustain professionalism. However, S-20 (Q-score = −4, z-score = −1.11), which advocates for leadership training and development programs, was ranked negatively, suggesting that respondents view such initiatives as insufficient without broader structural reforms. Additionally, the inclusion of governance frameworks that integrate climate resilience and disaster response received moderate support, as seen in S-41 (Q-score = 2, z-score = 0.61), indicating a growing recognition of sustainability considerations in governance reforms. This factor strongly prioritizes transparency, public accountability, and anti-corruption mechanisms, as evidenced by the highest positive z-scores for these elements. The negative z-scores assigned to statements that minimize accountability further reinforce the view that meaningful and sustainable public sector reforms remain unattainable without transparency measures. Factor 5 underscores the need for decentralization, anti-corruption efforts, and governance reforms to enhance public-sector efficiency. The findings suggest that while meritocratic principles and multi-stakeholder collaboration are critical to reform success, persistent political interference remains a formidable challenge. Although structural improvements and leadership training are acknowledged, the primary focus is on practical measures such as decentralization, strengthening accountability mechanisms, and mitigating political influence to ensure sustainable and effective public administration reforms in Bangladesh.

#### Factor 6: Reforming recruitment, promotion, and performance evaluation.

Factor 6, accounting for 5% of the explained variance and including eleven participants from diverse organizations, highlights the critical role of meritocracy, structured career advancement, and performance-based evaluation in fostering an efficient and accountable public sector. This factor underscores the necessity of transparent recruitment processes, competency-driven promotions, and professional development as key drivers of sustainable governance reforms. The highest-ranked statement, S-10 (Q-score = 5, z-score = 1.78), affirms that merit-based hiring and promotion systems are essential for mitigating nepotism and political patronage, underscoring the need for objective selection mechanisms. This perspective is further supported by S-8 (Q-score = 5, z-score = 1.51), which emphasizes the importance of transparent, competency-driven recruitment in enhancing the quality of public administration in Bangladesh. The role of structured career development and performance-based evaluation is also strongly affirmed. The positive rankings of S-20 (Q-score = 3, z-score = 1.12) and S-15 (Q-score = 2, z-score = 0.66) indicate that professional growth and promotion decisions should be based on merit and demonstrated contributions rather than tenure or political affiliations. However, political interference in recruitment and promotion processes remains a significant concern, as reflected in S-42 (Q-score = 4, z-score = 1.32) and S-14 (Q-score = 1, z-score = 0.26), highlighting that political favoritism continues to undermine the effectiveness of administrative reforms. While ethics-based governance and anti-corruption measures are recognized, they received lower rankings compared to structural recruitment and performance reforms. The negative rankings of S-3 (Q-score = −5, z-score = −2.06) and S-5 (Q-score = −3, z-score = −1.15) suggest skepticism about the effectiveness of ethical reforms alone without parallel improvements in recruitment and career progression frameworks. Similarly, professional development initiatives received mixed responses, as indicated by the neutral ranking of S-18 (Q-score = −1, z-score = −0.13), suggesting that professionalism is valued but may not be perceived as an isolated solution without institutional restructuring. Furthermore, innovation-driven governance approaches, such as S-23 (Q-score = −5, z-score = −2.42), were ranked negatively, indicating a preference for structured, rule-based professional development over flexible or adaptive reform models. This factor highlights systemic concerns in public sector human resource management, with strong positive z-scores assigned to statements advocating merit-based recruitment, competency-driven promotions, and performance-based evaluations. The rejection of subjective or politically influenced hiring and assessment processes further reinforces the preference for structured and transparent governance mechanisms. Factor 6 underscores that sustainable public sector reforms must prioritize depoliticized recruitment, meritocratic career progression, and institutionalized performance evaluations. While ethical concerns and anti-corruption measures remain relevant, structural improvements in hiring and evaluation processes are perceived as more effective for fostering accountability, efficiency, and long-term governance transformation.

### Analysis of methodological effectiveness on the Q method

Q methodology systematically examines subjectivity by integrating qualitative and quantitative methods, primarily through the Q-sort process, in which participants rank predefined statements along a forced distribution [42]. According to Ramlo [45] and Apine and Stojanovic [46], this method uses a small, purposive sample to reveal shared perspectives through factor analysis, emphasizing relational and comparative evaluation within an internally coherent framework, unlike traditional surveys. While the forced ranking offers notable insights in this study, it can complicate the interpretation of individual preferences outside the distribution [47]. Furthermore, post-sort interviews clarify reasoning, enhance interpretability and validity, and reduce bias, making Q methodology effective for capturing nuanced viewpoints without reducing them to rigid categories [46]. While valued for its structure and flexibility, Q methodology faces concerns over statement ambiguity. Still, its blend of qualitative depth and quantitative rigor makes it effective for capturing diverse subjective perspectives.

## Discussion

**Fig 3:**
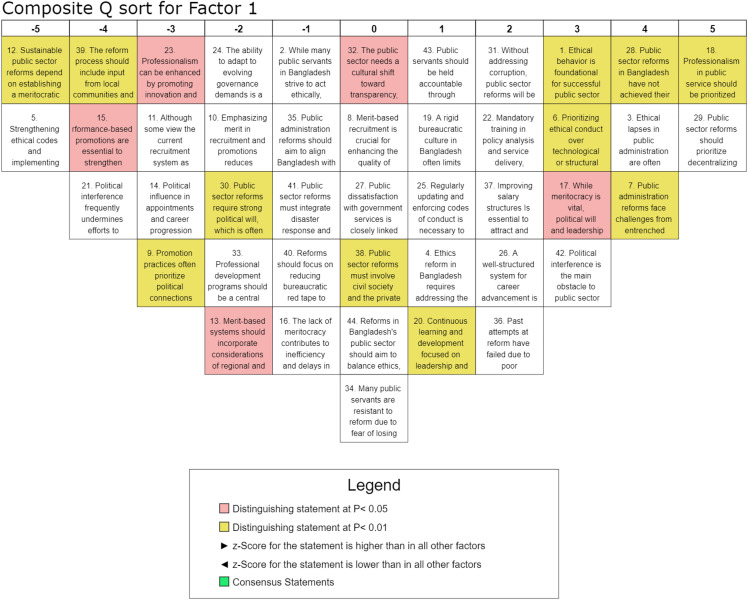
Composite analysis for Factor 1.

**Fig 4:**
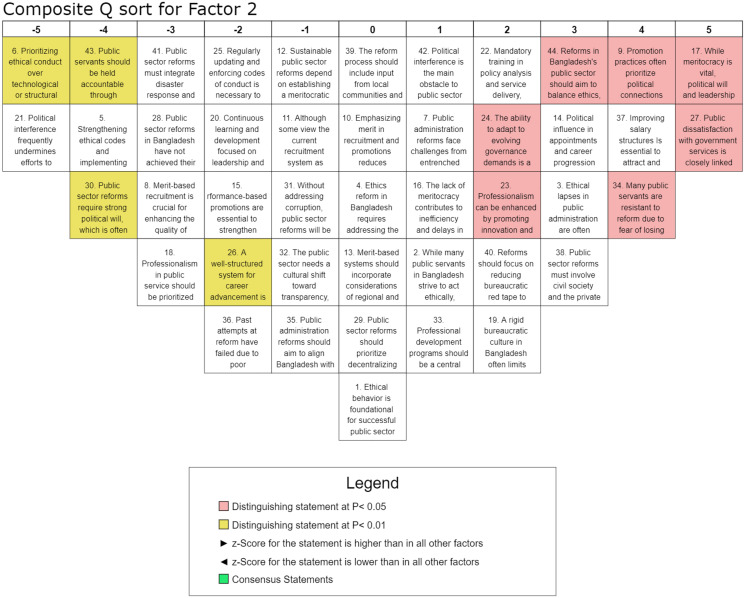
Composite analysis for Factor 2.

**Fig 5:**
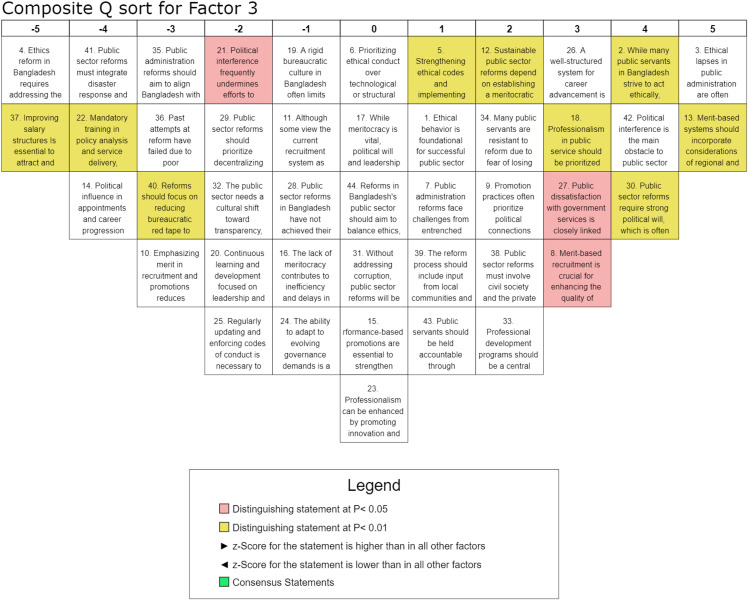
Composite analysis for Factor 3.

**Fig 6:**
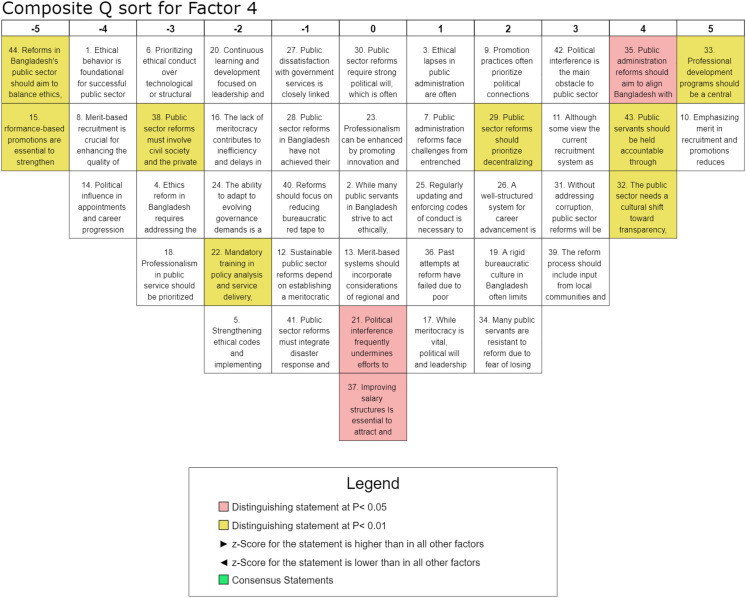
Composite analysis for Factor 4.

The study illustrates the complex nature of public sector reforms in Bangladesh, revealing six interrelated dimensions that encompass a) ethics, integrity, and accountability, b) political will and meritocracy, c) professional development, d) structural and institutional reforms, e) transparency and anti-corruption measures, and f) recruitment, promotion, and performance evaluation. These factors collectively emphasize the governance transformation and highlight the need for a cohesive, integrated approach to public sector reform. The findings align with the existing literature, reinforcing the holistic framework for achieving meaningful institutional improvements [22,48]. Despite broad consensus on key reform areas, Factor 1 sheds light on the pivotal role of ethics, integrity, and accountability in driving public sector reforms in Bangladesh, underscoring that ethical conduct is fundamental to meaningful governance transformation. As illustrated in Fig 3, the findings suggest that prioritizing ethical integrity over purely technological or structural interventions is essential for achieving sustainable and long-term reform outcomes. While meritocracy remains a fundamental pillar of public sector reform, its successful institutionalization is heavily contingent upon political will and leadership, as deeply embedded political interests and resistance to change often obstruct progress. Therefore, ensuring reform effectiveness requires comprehensive institutional safeguards and the implementation of depoliticized recruitment mechanisms to mitigate political interference [49]. The study emphasizes that continuous training and leadership development are necessary to enhance ethical decision-making, aligning with human capital theory and structured integrity programs, which have been shown to be effective in mitigating bureaucratic corruption globally [24]. Besides, public dissatisfaction with governance is linked to deficiencies in ethical conduct, reinforcing service motivation theory and highlighting the role of ethical commitment in service delivery. In this connection, strengthening transparency measures, regulatory oversight, and participatory governance is essential to restoring public trust [27]. Historically, past reform efforts have frequently failed due to poor implementation and institutional inertia, an outcome consistent with path dependency theory, highlighting the need for a multi-stakeholder approach involving civil society, the private sector, and international development partners to overcome established practices. These findings align with prior research by Andersson and Ekelund [30], reinforcing established insights and contributing to a broader understanding of the subject. The grid approach in Fig 3 highlights the robustness and relevance of the results within the existing body of literature. Henceforth, prioritizing professionalism alongside support for decentralized governance underscores the necessity of institutional integrity. At the same time, the limited emphasis on sanction-based measures highlights the importance of integrity-building strategies for sustainable, ethical reform, emphasizing the interdependence of moral integrity and bureaucratic efficiency as basis for transparent and accountable public administration. In Factor 2, as visualized in Fig 4, the interplay between political will, meritocracy, and accountability is highlighted in driving public sector reforms. As discussed earlier, strong political commitment is essential for effective governance, yet it remains fragile and is often undermined by political interference [50]. While meritocracy is crucial for improving service, entrenched political favoritism networks hinder progress, as public servants resist reforms due to fears of losing political favor. The study reinforces that reforms risk being ineffective without decisive political commitment, coined by Mulaphong [34]. Therefore, merit-based recruitment and performance-driven promotions are critical for enhancing efficiency in public sector. However, persistent political influence in appointments undermines these efforts, requiring governance reforms prioritizing transparency and ethical integrity [35]. Accountability is pivotal and requires strengthened ethical codes and practical administrative tools [20]. Also, continuous professional development is essential for fostering ethical behavior, innovation, and leadership within the public sector [39]. Despite the recognized need for reform, persistent barriers such as political interference, bureaucratic rigidity, and weak execution underline the importance of balancing ethical governance with administrative efficiency, streamlining bureaucratic procedures, and enhancing salary structures to attract and retain skilled professionals. It clarifies the urgency of reform and discloses that meaningful change hinges on tackling political and institutional challenges. Factor 3 participants highlight (Fig 5) the importance of professional development, leadership training, and ethical oversight in enhancing administrative efficiency, more than other factors. Studies by Geromichalos and Kospentaris [18] and Nasyrov [7] have underlined strong support for merit-based recruitment, which aligns with research on competency-based hiring as a driver of institutional effectiveness. Sustainable reforms require a meritocratic structure, strengthened ethical codes, and structured career advancement to improve governance [13]. However, political interference remains a significant barrier, undermining reform implementation and fostering resistance among public servants. Performance-based promotions and reduced bureaucratic red tape are crucial for attracting and retaining skilled professionals, consistent with the findings of Andersson and Ekelund [30]. While salary reforms are relevant, they are insufficient; continuous learning, leadership development, and accountability mechanisms are necessary. Although salary reforms are relevant, they are inadequate; sustained professional development, leadership training, and healthy accountability are essential for long-term effectiveness. Weak oversight structures further emphasize the need for reform frameworks. Ultimately, fostering a professional, transparent, and ethical public administration system is critical for achieving long-term reform success. Factor 4 emphasizes (Fig 6) the need for structural and institutional reforms, particularly administrative decentralization and streamlined governance, to enhance public-sector efficiency. This is consistent with the theoretical leans proposed by scholars such as Alam and Islam [1], who contend that sustainable improvements require integrating ethics, meritocracy, and decentralized governance to strengthen institutional effectiveness and accountability. The findings indicate that balancing ethical considerations with structural reforms is essential, alongside prioritizing professional development and career advancement to build capacity [23,51]. However, persistent political interference and patronage in recruitment hinder reform efforts. Decentralization and reducing bureaucratic inefficiencies are key recommendations, while mandatory policy analysis and service delivery training underscore the importance of capacity building [38]. Resistance to reform driven by political interests and dissatisfaction with public services underscores the need for a cultural shift toward transparency and accountability. The strong consensus on professionalization aligns with research on governance reforms. The strong consensus on the importance of professionalization aligns with existing research on governance reforms, reinforcing its role in enhancing public sector efficiency. This trend is consistent with the findings of Brinkerhoff and Brinkerhoff [15], who emphasize that professionalization and capacity-building initiatives are fundamental to effective governance transformations. Furthermore, the emphasis on aligning public administration reforms with the Sustainable Development Goals (SDGs) reflects adherence to global best practices in integrated governance, as outlined by Guterres [52] and Alam and Islam [1]. Institutional modernization, therefore, emerges as a critical imperative for achieving sustainable, long-term public sector reforms, requiring a comprehensive approach that integrates ethical governance, professional development, and structural efficiency.

**Fig 7:**
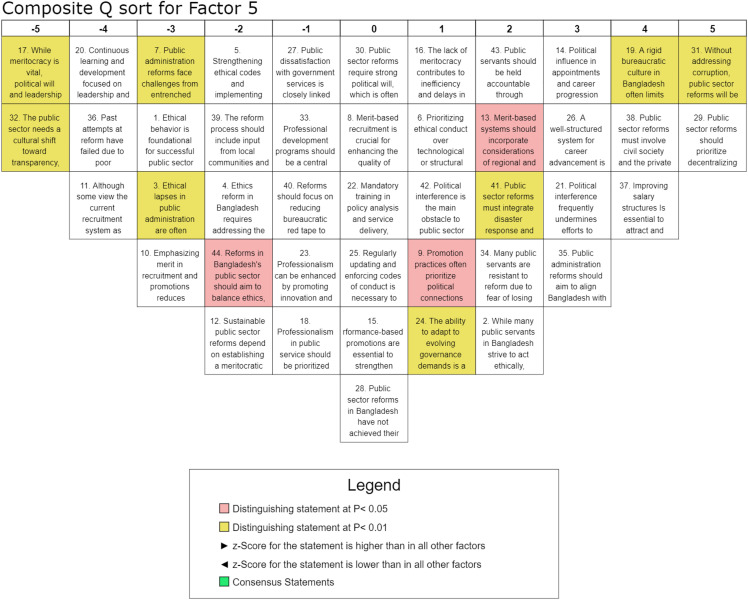
Composite analysis for Factor 5.

Factor 5 participants emphasize transparency and accountability as key drivers of successful public sector reform. As shown in Fig 7, their strong agreement that corruption obstructs governance improvements aligns with existing research demonstrating its detrimental effects on public trust and administrative efficiency [13]. Our findings align with studies by Nasyrov [7] and de Mattos Donadelli and Scott [22], showing that accountability, transparency, and anti-corruption measures are essential for effective reforms. However, ethical lapses and political interference in appointments hinder progress consistent with the arguments of Andersson and Ekelund [30]. Without targeted anti-corruption interventions, reforms risk ineffectiveness. Strengthening ethical codes, enforcing accountability mechanisms, and integrating disaster response strategies into reforms are critical for long-term sustainability [29]. Yet entrenched bureaucratic rigidity and public servants’ fear of losing benefits complicate reform efforts. Decentralization and participatory governance, supported by political will and leadership, can mitigate inefficiencies, according to Winsvold and Vabo [53]. Notably, respondents favor concrete policy interventions over abstract cultural changes, underscoring the need for action-oriented reforms rather than rhetorical commitments [39].

**Fig 8:**
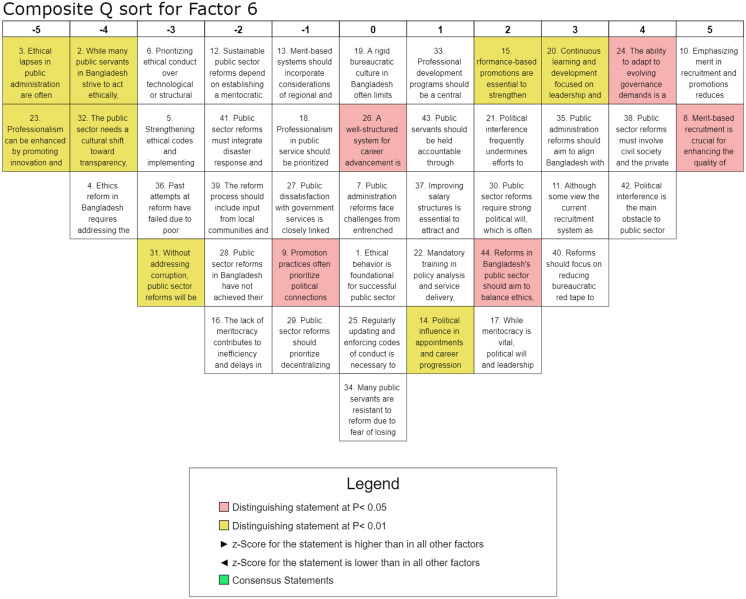
Composite analysis for Factor 6.

An effective public sector requires a transparent, merit-based recruitment and promotion system that strengthens public sector performance. Factor 6 visualizes (details in Fig 8) the significance of competency-driven hiring, performance-based promotions, and accountability in fostering administrative efficiency. Our findings highlight that merit-based recruitment enhances service quality, while performance-driven promotions reinforce institutional integrity [34]. However, political interference in appointments and career progression undermines equitable advancement, contributing to inefficiency and delays [3], as Eryanto et al. [9] mentioned. Meanwhile, without addressing corruption, public sector reforms will remain ineffective. A structured career advancement framework, continuous professional development, and the cultivation of ethical leadership are essential for sustaining governance improvements [25,27]. In addition, bureaucratic inefficiencies and ethical lapses necessitate stronger accountability measures [30]. Respondents overwhelmingly support depoliticized hiring as the key to administrative professionalism [35], emphasizing that governance reforms must curb political favoritism [50]. Thus, reforms must integrate transparency, meritocracy, and structural improvements to enhance the effectiveness of recruitment, promotion, and performance evaluation processes.

## Implications and contributions

### Theoretical contribution

This study offers key theoretical contributions to public administration and governance, particularly in developing countries such as Bangladesh. First, it advances the theoretical discourse on ethical governance by showing that corrective actions alone are insufficient to foster professionalism, highlighting the need for institutionalized ethical training. This aligns with institutional theory [54], which argues that formal rules must be supported by normative and cognitive instruments to shape individual and organizational ethics. Second, this study extends the public choice theory [55] by showing how strong political will can mitigate patronage and nepotism, enabling meritocratic civil service reforms. It challenges the assumption that political actors always act in their own self-interest, highlighting leadership’s potential to foster a more impartial and effective bureaucracy [27]. Third, by highlighting the role of professional development and competency-based recruitment, this study contributes to human capital theory [56], which posits that investments in education, training, and leadership development enhance productivity and efficiency. The findings underscore that a well-trained civil service, supported by continuous learning and leadership programs, leads to improved governance outcomes and more effective policy implementation. Furthermore, the study provides empirical support for structural contingency theory [57], which suggests that organizational effectiveness depends on aligning governance structures with environmental demands. The research demonstrates that decentralization, process streamlining, and alignment with international best practices, such as the SDGs, contribute to the adaptability and efficiency of public sector institutions. This research deepens the understanding of how to optimize governance structures to address institutional challenges in developing countries. Moreover, the study extends the agency theory [58] in the public sector by examining how anti-corruption and transparency measures enhance accountability. By advocating for data-driven oversight, participatory governance, and digital transparency, the research highlights how reducing information asymmetry and strengthening monitoring can limit bureaucratic inefficiencies and corrupt practices. Lastly, this research contributes to meritocratic governance by reinforcing that transparent recruitment processes and structured performance evaluation frameworks are fundamental to enhancing bureaucratic efficiency [18]. The study’s findings provide evidence supporting theoretical claims about the need to reduce political interference in hiring and promotion decisions to build a more competent and impartial public administration. It enriches academic debates on governance reforms by integrating insights from theoretical contributions. Also, it provides a contextualized understanding of how institutional, political, and structural insights can advance governance reform in developing countries.

### Policy implications for public sector reforms

The study reveals key policy implications for public-sector reform in Bangladesh, emphasizing the need for inclusive governance strategies to address systemic inefficiencies. Strengthening ethical governance is fundamental, requiring rigorous ethical training programs and extensive enforcement mechanisms that promote integrity within the civil service. Moving beyond retaliatory measures, fostering a culture of ethical accountability through continuous training, mentoring, and institutionalized codes of conduct is essential. Political commitment is a prerequisite for sustainable reform. Subsequently, institutionalizing meritocratic principles necessitates strong political leadership to eliminate entrenched patronage and nepotism, ensuring impartiality in recruitment and promotions. Legislative and institutional safeguards must reinforce decision-making processes to prevent political interference in civil service appointments. Furthermore, investing in professional development is critical for building a competent and future bureaucracy. In line with this, continuous training programs, leadership development initiatives, and competency-based recruitment bases should align with evolving governance challenges to enhance policy execution and service delivery. Structural and institutional overhauls are necessary to modernize public administration and improve governance outcomes. Decentralization, streamlined bureaucratic processes, and alignment with international best practices, such as the SDGs, are vital for increasing the efficiency and adaptability of government institutions. Institutional restructuring should minimize red tape, promote inter-agency coordination, and encourage evidence-based policymaking. Strengthening anti-corruption and transparency measures is imperative for ensuring accountability. Also, it needs participatory governance frameworks, independent oversight bodies, and data-driven monitoring systems to enhance corruption detection and prevention. Digital governance tools such as e-governance platforms and automated decision-making systems can strengthen transparency and reduce discretionary abuse. Finally, merit-based recruitment and transparent performance evaluations can reduce political influence and promote professionalism. Together, these reforms will create an effective, accountable, and resilient public administration aligned with Bangladesh’s development goals.

## Limitations and future research avenues

This study examines the roles of ethics, meritocracy, and professionalism in public sector reforms, offering critical insights into the transformation of governance. However, several limitations should be acknowledged. First, while the research supports the conceptual framework for public sector reforms, it relies primarily on qualitative insights, which may limit its empirical robustness. Future studies should adopt mixed-method approaches that incorporate quantitative analysis to measure the impact of ethics, meritocracy, and professionalism on governance efficiency. Second, although the study includes diverse participants from the public sector, it is limited to a specific administrative context, which restricts its applicability to broader governance systems. Third, the study does not examine how the formation of professional identity influences administrative practices, thereby strengthening the theoretical framework for ethical governance. Additionally, while the study identifies political interference and patronage networks as significant barriers, it does not thoroughly examine the underlying power dynamics that sustain these practices, indicating a need for further political economy analyses. Future research should address these limitations by exploring several key areas. Longitudinal studies are essential to assess the long-term impacts of ethics-driven and meritocratic reforms in public administration, as they would provide more substantial empirical evidence for policy recommendations. Additionally, future studies should adapt and expand data-collection frameworks to accommodate diverse institutional and cultural contexts, enabling comparative research across various governance systems. Employing Q methodology could capture diverse stakeholder perspectives, thereby enhancing cross-contextual governance analysis. Moreover, research should investigate the effectiveness of professional development programs and leadership training in promoting ethical decision-making, reducing corruption, and improving bureaucratic efficiency. The potential of emerging digital governance tools, e.g., artificial intelligence, blockchain, and e-governance, should also be explored to address bureaucratic inefficiencies and political interference. These technological interventions may offer alternative mechanisms to enhance transparency, accountability, and ethical governance. Finally, further research should analyze the nexus between ethical governance, institutional reforms, and political leadership to develop actionable strategies for policymakers. Also, investigating governance structures that minimize political interference while incentivizing professional integrity could provide a comprehensive framework for sustainable public sector reforms.

## Conclusion

This study utilizes Q methodology to examine the roles of ethics, meritocracy, and professionalism in public sector reforms, revealing diverse perspectives on reform priorities. The findings underscore the complex nature of reform efforts, as various stakeholder groups prioritize distinct but interconnected aspects of change. Key drivers for effective public sector transformation include ethical governance, merit-based systems, and the reduction of bureaucratic inefficiencies. Participants in Factor 1 emphasize the critical role of ethics and accountability, arguing that reform initiatives will fail without addressing corruption. Factor 2 highlights the need for strong political will to reduce political interference in appointments and decision-making. Factor 3 highlights the importance of institutionalizing meritocracy and professionalism through structured career pathways, and Factor 4 calls for inclusive reform strategies that engage civil society and private-sector collaboration to improve governance outcomes. Factor 5 recognizes ongoing challenges in reform implementation stemming from institutional inertia and resistance to change. Finally, Factor 6 emphasizes the need for continuous learning and leadership development to ensure long-term administrative efficiency. The findings indicate that successful public sector reforms require an integrated approach that combines ethical governance, merit-based recruitment, and professional capacity building, while addressing bureaucratic inefficiencies and political constraints. Future research should investigate the interactions among these dimensions in different governance contexts to support the development of sustainable and effective reform strategies.

## Supporting information

S1 File **S1 Table.** List all Q-sample statements used in the Q methodology process. **S2 Table.** Factor loading for each factor and participants with flagged sorts are marked in bold. **S1 Data.**
(ZIP)
