# Supplementary material for: Role of ethics, meritocracy, and professionalism in public sector reforms: A Q methodology study
Source: PLoS One. 2026 Feb 20;21(2):e0342981. doi: 10.1371/journal.pone.0342981 (PMC12923134; doi:10.1371/journal.pone.0342981)
Supplement: S1 File — S1 Table. List all Q-sample statements used in the Q methodology process. S2 Table. Factor loading for each factor and participants with flagged sorts are marked in bold. S1 Data. (ZIP) [file pone.0342981.s001.zip › S1 Table.docx]

**Table SI** List all Q-sample statements used in the Q methodology process.

Please rate each statement below based on how closely it aligns with your opinion, using the following scale from +5 to -5: (-5: Least like my opinion, -4: Strongly disagree, -3: Disagree: -2: Slightly disagree: -1: Mildly disagree, 0: Neutral, +1: Mildly agree +2 Slightly agree: +3: Agree +4: Strongly agree, +5 Most like my opinion). This structure allows respondents to understand the gradation of opinion, from disagreement (-5) to agreement (+5), with a neutral midpoint at 0.

| **SI** | **Statements** |
| --- | --- |
| **A** | **Ethics in Public Sector Reforms** |
| 1 | Ethical behavior is foundational for successful public sector reforms, as it promotes fair resource allocation and mitigates discrimination. |
| 2 | While many public servants in Bangladesh strive to act ethically, systemic issues and a lack of ethics training often hinder their efforts, contributing to inefficiencies in service delivery. |
| 3 | Ethical lapses in public administration are often ignored due to weak oversight mechanisms, and existing codes of ethics are frequently symbolic without adequate enforcement. |
| 4 | Ethics reform in Bangladesh requires addressing the deep-rooted causes of corruption that pose significant challenges to public sector reforms. |
| 5 | Strengthening ethical codes and implementing harsher consequences for violations could enhance transparency and accountability. |
| 6 | Prioritizing ethical conduct over technological or structural reforms may be essential for meaningful change in governance. |
| 7 | Public administration reforms face challenges from entrenched corruption and are difficult to change through reform. |
| **B** | **Meritocracy in Public Sector Reforms** |
| 8 | Merit-based recruitment is crucial for enhancing the quality of public administration in Bangladesh. |
| 9 | Promotion practices often prioritize political connections over merit, undermining reform efforts. |
| 10 | Emphasizing merit in recruitment and promotions reduces nepotism and fosters accountability. |
| 11 | Although some view the current recruitment system as transparent, reforms are needed to ensure genuine meritocracy. |
| 12 | Sustainable public sector reforms depend on establishing a meritocratic foundation. |
| 13 | Merit-based systems should incorporate considerations of regional and social diversity to enhance inclusivity. |
| 14 | Political influence in appointments and career progression deters qualified professionals, diminishing reform impacts. |
| 15 | Performance-based promotions are essential to strengthen professionalism over tenure-based advancement. |
| 16 | The lack of meritocracy contributes to inefficiency and delays in achieving reform goals. |
| 17 | While meritocracy is vital, political will and leadership are necessary to drive meaningful reforms in public administration. |
| **C** | **Professionalism in Public Sector Reforms** |
| 18 | Professionalism in public service should be prioritized over political loyalty to improve public administration quality. |
| 19 | A rigid bureaucratic culture in Bangladesh often limits skill development, leaving many public servants without essential professional competencies. |
| 20 | Continuous learning and development focused on leadership and management skills are essential for fostering professionalism in the public sector. |
| 21 | Political interference frequently undermines efforts to cultivate professionalism among public servants. |
| 22 | Mandatory training in policy analysis and service delivery, emphasizing strategic thinking and professional conduct, should be instituted for public servants. |
| 23 | Professionalism can be enhanced by promoting innovation and adaptability within public sector roles. |
| 24 | The ability to adapt to evolving governance demands is a key measure of professionalism in public administration. |
| 25 | Regularly updating and enforcing codes of conduct is necessary to maintain and strengthen professionalism. |
| 26 | A well-structured system for career advancement is essential for sustaining professionalism within public administration. |
| 27 | Public dissatisfaction with government services is closely linked to a lack of professionalism, which requires addressing the deep-seated patronage system and enhancing ethical standards, accountability, and fair compensation. |
| **D** | **General Opinions about Public Sector Reform** |
| 28 | Public sector reforms in Bangladesh have not achieved their intended outcomes due to political challenges. |
| 29 | Public sector reforms should prioritize decentralizing power to local governments to improve public service delivery and better management of public resources. |
| 30 | Public sector reforms require strong political will, which is often lacking. |
| 31 | Without addressing corruption, public sector reforms will be ineffective. |
| 32 | The public sector needs a cultural shift toward transparency, accountability, and human resource development to enable meaningful reforms. |
| 33 | Professional development programs should be a central part of any reform effort. |
| 34 | Many public servants are resistant to reform due to fear of losing privileges. |
| 35 | Public administration reforms should aim to align Bangladesh with the Sustainable Development Goals (SDGs). |
| 36 | Past attempts at reform have failed due to poor coordination among ministries. |
| 37 | Improving salary structures is essential to attract and retain skilled professionals in the public sector. |
| 38 | Public sector reforms must involve civil society and the private sector for holistic change. |
| 39 | The reform process should include input from local communities and citizens to ensure effectiveness. |
| 40 | Reforms should focus on reducing bureaucratic red tape to improve efficiency. |
| 41 | Public sector reforms must integrate disaster response and climate resilience strategies. |
| 42 | Political interference is the main obstacle to public sector reform in Bangladesh. |
| 43 | Public servants should be held accountable through regular, independent evaluations. |
| 44 | Reforms in Bangladesh's public sector should aim to balance ethics, meritocracy, and professionalism. |
