# Supplementary material for: Role of ethics, meritocracy, and professionalism in public sector reforms: A Q methodology study
Source: PLoS One. 2026 Feb 20;21(2):e0342981. doi: 10.1371/journal.pone.0342981 (PMC12923134; doi:10.1371/journal.pone.0342981)
Supplement: S1 File — S1 Table. List all Q-sample statements used in the Q methodology process. S2 Table. Factor loading for each factor and participants with flagged sorts are marked in bold. S1 Data. (ZIP) [file pone.0342981.s001.zip › S2 Table.docx]

**Table S2** Factor loading for each factor and participants with flagged sorts are marked in bold

| **Q sort ID** | **Factor 1** | **Factor 2** | **Factor 3** | **Factor 4** | **Factor 5** | **Factor 6** |
| --- | --- | --- | --- | --- | --- | --- |
| 1 | **0.4180** | -0.0314 | -0.1191 | -0.1389 | -0.0934 | 0.2451 |
| 2 | -0.4587 | 0.3202 | -0.1011 | 0.2004 | 0.1785 | 0.1779 |
| 3 | -0.5019 | 0.4817 | -0.1533 | -0.0745 | -0.0885 | -0.0283 |
| 4 | 0.0194 | -0.1097 | **0.7315** | 0.0595 | 0.1357 | -0.0650 |
| 5 | -0.0251 | 0.0418 | -0.0968 | **0.6479** | -0.0614 | -0.0578 |
| 6 | **0.4744** | -0.0059 | -0.1110 | 0.1329 | -0.2053 | 0.2294 |
| 7 | 0.2697 | 0.3369 | -0.3534 | 0.1983 | 0.2635 | 0.0417 |
| 8 | -0.1835 | 0.2105 | -0.1165 | -0.0725 | -0.0994 | **-0.4534** |
| 9 | 0.0729 | 0.0212 | **0.5886** | 0.0056 | -0.0336 | 0.1017 |
| 10 | -0.0122 | 0.2087 | **0.7159** | 0.1388 | -0.1033 | -0.0027 |
| 11 | 0.0055 | -0.0139 | **-0.1801** | -0.0478 | **-0.3661** | 0.1445 |
| 12 | 0.2995 | 0.1011 | 0.3611 | -0.1525 | 0.0746 | -0.2196 |
| 13 | 0.0875 | 0.1290 | -0.174 | -0.2091 | -0.3300 | -0.1178 |
| 14 | **0.2587** | 0.0311 | 0.1205 | 0.0773 | 0.0539 | 0.0081 |
| 15 | **0.4145** | -0.1048 | -0.2945 | 0.1188 | 0.0560 | -0.2786 |
| 16 | -0.0883 | 0.2505 | 0.1009 | **0.4890** | 0.1493 | 0.0466 |
| 17 | -0.1517 | -0.0667 | 0.0675 | -0.0371 | **-0.6106** | -0.1311 |
| 18 | **0.4153** | 0.3831 | -0.0894 | 0.0549 | 0.1527 | 0.0420 |
| 19 | -0.0702 | -0.2247 | 0.2998 | -0.2109 | 0.1013 | -0.0608 |
| 20 | -0.0065 | 0.0634 | -0.1674 | -0.0567 | 0.1750 | **0.2680** |
| 21 | 0.2191 | 0.0739 | 0.0798 | **0.6293** | -0.0438 | -0.0626 |
| 22 | 0.1591 | -0.1749 | -0.2833 | 0.5094 | 0.1130 | 0.2335 |
| 23 | 0.2526 | **0.5915** | 0.0114 | 0.0255 | -0.0117 | -0.0304 |
| 24 | 0.1070 | 0.1033 | 0.3251 | -0.4058 | -0.3004 | 0.0694 |
| 25 | 0.0715 | 0.1325 | -0.1091 | 0.0087 | 0.1576 | -0.2756 |
| 26 | -0.1760 | -0.1369 | -0.1689 | 0.2652 | **0.5362** | -0.1463 |
| 27 | -0.0592 | 0.0659 | **-0.4637** | -0.1674 | 0.2950 | -0.0471 |
| 28 | -0.0589 | **0.5508** | 0.0068 | 0.1703 | 0.0676 | 0.0237 |
| 29 | -0.0948 | -0.2146 | 0.0933 | -0.1211 | 0.1760 | **0.6862** |
| 30 | 0.0744 | -0.4008 | -0.0904 | -0.2179 | -0.4478 | 0.0685 |
| 31 | 0.0913 | 0.1516 | -0.0849 | 0.1082 | -0.2703 | **0.4249** |
| 32 | 0.3191 | -0.0561 | -0.1186 | -0.1369 | 0.1527 | -0.3515 |
| 33 | -0.0292 | 0.1275 | -0.0565 | 0.0819 | -0.0225 | **0.4940** |
| 34 | 0.2061 | 0.1780 | 0.0621 | **0.3307** | -0.2085 | -0.1359 |
| 35 | 0.1706 | -0.2245 | **0.3153** | 0.3011 | -0.1332 | -0.2813 |
| 36 | -0.4011 | 0.1721 | -0.0974 | -0.3753 | -0.0352 | -0.1142 |
| 37 | -0.0068 | -0.2215 | -0.2377 | **-0.3679** | -0.2487 | 0.1057 |
| 38 | -0.3895 | -0.2078 | -0.0898 | 0.2162 | 0.2742 | 0.2639 |
| 39 | 0.2929 | 0.0380 | -0.0357 | 0.0127 | **-0.3022** | 0.0487 |
| 40 | 0.2307 | 0.0565 | -0.1636 | **-0.4225** | 0.2874 | -0.2692 |
| 41 | -0.2670 | -0.3105 | -0.3084 | 0.0927 | -0.1792 | -0.0018 |
| 42 | -0.0855 | 0.1326 | 0.1383 | -0.0240 | 0.1573 | **0.4125** |
| 43 | **0.4835** | -0.0962 | -0.0141 | -0.3436 | -0.0887 | -0.3054 |
| 44 | 0.1581 | 0.2247 | -0.2642 | -0.2077 | **0.4304** | 0.0362 |
| 45 | -0.1584 | 0.1539 | -0.4172 | 0.1819 | -0.4184 | -0.2333 |
| 46 | -0.1508 | **0.6203** | -0.0227 | 0.0953 | 0.0079 | 0.0067 |
| 47 | -0.1030 | 0.2940 | 0.0369 | -0.1891 | -0.0389 | **0.3250** |
| 48 | 0.2492 | -0.2084 | -0.1675 | 0.0635 | **0.3994** | 0.0255 |
| 49 | -0.1332 | 0.1900 | **0.3979** | -0.0257 | 0.3911 | -0.0881 |
| 50 | -0.0322 | 0.1511 | -0.1943 | 0.1686 | -0.0262 | -0.0111 |
| 51 | 0.1350 | 0.0360 | 0.0798 | 0.3135 | -0.2579 | **-0.3205** |
| 52 | -0.1202 | 0.1544 | -0.3181 | 0.0071 | -0.0411 | -0.2899 |
| 53 | 0.1018 | -0.0422 | -0.0413 | -0.0541 | **0.4895** | 0.1663 |
| 54 | **0.6554** | -0.0757 | 0.1827 | -0.0233 | 0.1045 | -0.2039 |
| 55 | **0.6200** | 0.1568 | 0.0175 | 0.2293 | 0.1465 | -0.0893 |
| 56 | -0.0284 | -0.1044 | -0.2713 | 0.2256 | 0.3355 | 0.2111 |
| 57 | 0.0120 | **-0.5384** | -0.0823 | 0.1426 | 0.0614 | -0.0067 |
| 58 | 0.3216 | 0.0934 | -0.1551 | -0.2339 | 0.0562 | 0.5011 |
| 59 | -0.1344 | -0.1675 | 0.3258 | -0.0001 | 0.0945 | **0.4063** |
| 60 | -0.0267 | **-0.5817** | 0.1343 | 0.0732 | 0.1008 | -0.0436 |
| 61 | -0.2349 | 0.1918 | -0.1432 | 0.1806 | -0.0703 | -0.0952 |
| 62 | -0.0865 | 0.2080 | 0.0436 | 0.1234 | **-0.2956** | 0.0820 |
| 63 | 0.1098 | 0.4755 | 0.0182 | **-0.6161** | -0.0444 | -0.1158 |
| 64 | -0.1427 | -0.0360 | -0.0183 | 0.0009 | -0.0592 | 0.1616 |
| 65 | 0.2604 | 0.2618 | 0.1268 | -0.2801 | 0.0338 | 0.2057 |
| 66 | -0.2216 | 0.2551 | 0.2029 | -0.2157 | **0.3607** | 0.2284 |
| 67 | 0.0827 | 0.1377 | -0.1652 | 0.0833 | -0.1888 | **0.5560** |
| 68 | 0.0614 | 0.1448 | 0.2066 | -0.0892 | **0.4983** | -0.1535 |
